# MiRNAs and LncRNAs: Dual Roles in TGF-β Signaling-Regulated Metastasis in Lung Cancer

**DOI:** 10.3390/ijms21041193

**Published:** 2020-02-11

**Authors:** Xing-Ning Lai, Jun Li, Li-Bo Tang, Wen-Tong Chen, Lei Zhang, Li-Xia Xiong

**Affiliations:** Department of Pathophysiology, Jiangxi Province Key Laboratory of Tumor Pathogenesis and Molecular Pathology, Medical College, Nanchang University, 461 Bayi Road, Nanchang 330006, China; laixingning99@outlook.com (X.-N.L.); lj012729@163.com (J.L.); lb525912836@163.com (L.-B.T.); c0813408@163.com (W.-T.C.); zhangleihen6@163.com (L.Z.)

**Keywords:** miRNAs, lncRNAs, TGF-β signaling, miRNA sponges, lung cancer, metastasis

## Abstract

Lung cancer is one of the most malignant cancers around the world, with high morbidity and mortality. Metastasis is the leading cause of lung cancer deaths and treatment failure. MicroRNAs (miRNAs) and long non-coding RNAs (lncRNAs), two groups of small non-coding RNAs (nc-RNAs), are confirmed to be lung cancer oncogenes or suppressors. Transforming growth factor-β (TGF-β) critically regulates lung cancer metastasis. In this review, we summarize the dual roles of miRNAs and lncRNAs in TGF-β signaling-regulated lung cancer epithelial-mesenchymal transition (EMT), invasion, migration, stemness, and metastasis. In addition, lncRNAs, competing endogenous RNAs (ceRNAs), and circular RNAs (circRNAs) can act as miRNA sponges to suppress miRNAs, thereby mediating TGF-β signaling-regulated lung cancer invasion, migration, and metastasis. Through this review, we hope to cast light on the regulatory mechanisms of miRNAs and lncRNAs in TGF-β signaling-regulated lung cancer metastasis and provide new insights for lung cancer treatment.

## 1. Introduction

Lung cancer is the most common cancer based on current diagnoses and the leading cause of cancer-related deaths globally [1]. According to the data from the GLOBOCAN database, it is estimated that 2.09 million new cases (11.6% of total cancer cases) and a mortality of 1.76 million (18.4% of total cancer-related deaths) of lung cancer were found worldwide in 2018 [2]. Lung cancer can be divided into small cell lung cancer (SCLC) (15%) and non-small cell lung cancer (NSCLC) (85%). NSCLC contains three major subtypes: lung adenocarcinoma (40%), lung squamous cell carcinoma (25–30%), and large cell carcinoma (10–15%) [3,4]. Lung cancer development is mostly correlated with genetic mutations and environmental interactions, such as cigarette smoking, radiation exposure, and toxic substances [5]. Presently, metastasis and recurrence are the main causes of death for lung cancer patients [6]. Statistics show that approximately two-thirds of patients have metastatic lesions when they are first diagnosed with lung cancer [7]. Although there are many therapies (surgical resection, chemotherapy, radiotherapy, etc.) to treat lung cancer, the overall five-year survival rate of lung cancer patients is still as low as 17.7% [8], and the overall five-year survival rate of patients with distant organ metastasis is 4.5% [9]. Therefore, it is necessary to explore the metastatic mechanisms of lung cancer, as finding new strategies to diagnose and treat lung cancer is of great significance.

MicroRNAs (miRNAs) and long non-coding RNAs (lncRNAs) are small non-coding RNAs (nc-RNAs) without the potential to encode proteins [10]. Nevertheless, these RNAs can regulate gene transcription and translation by interacting with the 3′ untranslated region (3′UTR) of messenger RNA (mRNA) [11,12]. Functionally, they can regulate various cellular processes, such as cell proliferation, differentiation, and apoptosis [13,14]. Further, these two RNAs have been found to play anti-carcinogenic or oncogenic roles in lung cancer progression, and their aberrant expressions can give rise to the malignant developments of lung cancer, such as migration, invasion, and metastasis [15]. Additionally, miRNAs and lncRNAs can also be used as biomarkers to predict the outcomes of lung cancer patients [16]. Accumulating reports have found that both lncRNAs and miRNAs can regulate the transforming growth factor-β (TGF-β) signaling pathways, thus affecting lung tumor migration, invasion, and metastasis [17,18]. Some lncRNAs can modulate the TGF-β-induced epithelial-mesenchymal transition (EMT) by interacting with miRNAs [19,20]. TGF-β, a multifunctional cytokine, has three isoforms, TGF-β1, TGF-β2, and TGF-β3 [21]. It can usually potentiate migration, invasion, and metastasis in lung cancer [22].

In this review, we summarize the positive and negative roles of lncRNAs and miRNAs in TGF-β signaling-regulated lung cancer metastasis, hoping to provide new insights for the design of more efficient therapies for lung cancer treatment in the future.

## 2. Overview of miRNAs and lncRNAs

### 2.1. MiRNAs

MiRNAs, 18~25 nucleotides in length, can bind to miRNA response elements (MREs) on the target mRNA 3′UTR and silence target genes through mRNA degradation or by suppressing mRNA translation [23]. Notably, each miRNA can target various mRNAs, thereby mediating several genes; each gene can also be regulated by multiple miRNAs [24]. At present, miRNAs are attracting increasingly more attention due to the fact that their dysregulation plays a critical role in many human diseases, especially cancers [25]. Abnormal miRNA expression is always associated with lung cancer progression [26]. Furthermore, miRNAs can cause resistance to drugs and radio-chemotherapy in lung cancer patients, contributing to low therapeutic efficiency [27]. They can also be predictors for lung cancer prognosis [28].

### 2.2. LncRNAs

LncRNAs are another class of nc-RNAs with more than 200 nucleotides in length. In the cell nucleus, lncRNAs can modify chromatin structures by interacting with polycomb repressive complex 2 (PRC2), enhance or reduce gene transcription by recruiting transcription factors, and regulate miRNA processing [29]. In the cytoplasm, lncRNAs can mediate mRNA translation, increase or decrease mRNA stability, and act as miRNA sponges that can repress miRNAs [29,30]. LncRNAs can participate in the pathogenesis of various human diseases, particularly cancers [31]. LncRNAs have similar carcinogenic and anti-carcinogenic capacities to miRNAs, affecting lung cancer proliferation, migration, invasion, and metastasis [32]. Particularly, lncRNAs play a critical role in drug resistance. Targeting lncRNAs may help increase drug sensibility during lung cancer treatments [33].

## 3. TGF-β Signaling Is Involved in Lung Cancer Metastasis

### 3.1. TGF-β Signaling Regulates Cell Migration and Invasion

**Figure 1:**
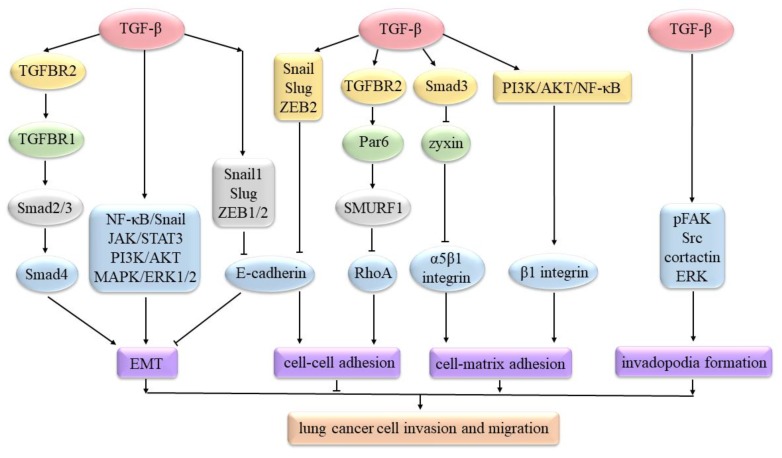
TGF-β signaling regulates lung cancer cell invasion and migration. TGF-β can mediate EMT via Smad pathways and non-Smad pathways. TGF-β can increase Snail, Slug, and ZEB2 expression to decrease E-cadherin. It can also bind to TGFBR2 and then phosphorylate Par6 to recruit Smad ubiquitin regulatory factor 1 (SMURF1). SMURF1 can degrade RhoA. These can reduce cell–cell adhesion. TGF-β1 can activate PI3K/AKT/NF-κB to increase β1 integrin expression. TGF-β/Smad3 signal can increase α5β1 integrin expression by down-regulating zyxin. These can enhance cell–matrix adhesion. TGF-β can increase phosphorylated focal adhesion kinase (pFAK), Src, cortactin, and ERK in response to β3 integrin, resulting in forming a mature invadopodia.

TGF-β mediates the EMT process mostly via the Smad (Figure 1) pathways. In this model, activated TGF-β first binds to TGF-β receptor 2 (TGFBR2), which can recruit and then phosphorylate TGFBR1. Subsequently, phosphorylated TGFBR1 activates Smad2 and Smad3. Smad2/3 can bind to Smad4 and enter into the cell nucleus to regulate the expression of EMT-associated genes or factors [34]. TGF-β can increase the expression of EMT-inducing transcription factors, such as Snail1, Slug (Snail2), and Zinc finger E-box-binding homeobox 1 and 2 (ZEB1/2) [35]. These transcription factors can repress E-cadherin expression to induce EMT in lung cancer [36]. TGF-β can also function through non-Smad (Figure 1) pathways to modulate lung cancer EMT, such as the nuclear factor-kappa B (NF-κB)/Snail [37], Janus-activated kinase (JAK)/signal transducer and activator of transcription 3 (STAT3) [38], phosphatidylinositol-3-kinase (PI3K)/protein kinase B (AKT), and mitogen-activated protein kinase (MAPK)/extracellular signal-regulated kinase 1/2 (ERK1/2) [39] pathways.

TGF-β can induce Snail, Slug, and ZEB2 expression to decrease E-cadherin, resulting in breaking adherens junctions [40]. TGF-β can bind to TGFBR2 and then phosphorylate Par6 to recruit SMURF1. Then, SMURF1 can degrade RhoA to increase the dissolution of junctional complexes and thereby reduce cell–cell adhesions [41]. In contrast to cell–cell adhesions, cell–matrix adhesions are always enhanced in lung cancers (Figure 1). In A549 cells, TGF-β1 can activate the PI3K/AKT/NF-κB signaling pathway to increase β1 integrin expression, which may facilitate integrin-mediated cell migration [42]. TGF-β/Smad3 signal can increase α5β1 integrin expression by down-regulating zyxin. Up-regulated α5β1 integrin can enhance A549 cell adhesion to the fibronectin, as well as increase migration [43].

Invadopodia can degrade the surrounding extracellular matrix (ECM) through matrix-degrading proteinases, such as membrane type 1 matrix metalloprotease (MT1-MMP), MMP2, MMP9, cathepsin B, and a disintegrin and metalloprotease 12 (ADAM12), thus propelling cell invasion [44,45]. In H157 cells, TGF-β can increase pFAK, Src, cortactin, and ERK in response to β3 integrin, eventually resulting in forming a mature invadopodia with proteolytic activity (Figure 1) [46].

### 3.2. TGF-β Signaling Regulates Lung Cancer’s Distant Metastasis

Lung tumor cell intravasation is involved in migrating to and then passing through the vessel wall. Tumor cells not only secrete TGF-β1 but also bind to Notch ligands on endothelial cells (ECs) by using Notch receptors, thus facilitating their transmigration through the endothelial tight junctions [47]. TGF-β can increase β3 integrin expression to enhance H157 cell adhesion to lymphatic endothelial cells (LECs), subsequently promoting cell transmigration across LEC monolayers [48]. The consequences of successful intravasation are indicated by the appearance of circulating tumor cells (CTCs) in circulation.

**Figure 2:**
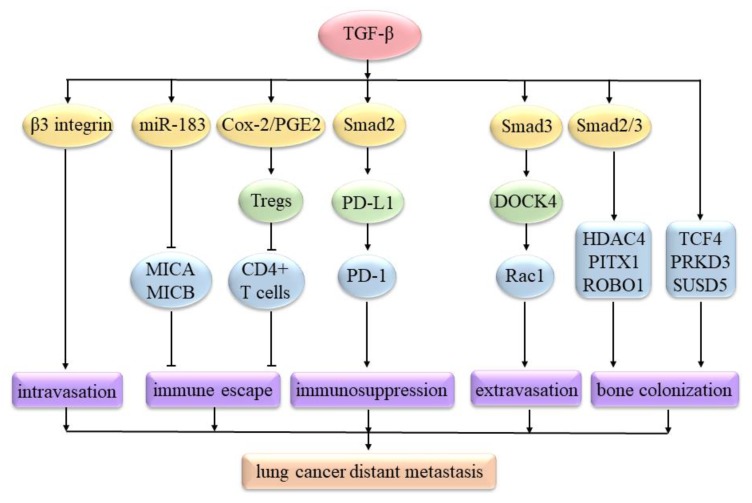
TGF-β signaling regulates lung cancer’s distant metastasis. TGF-β can increase β3 integrin expression to enhance cancer cell intravasation. TGF-β can increase Tregs via Cox-2/PGE2 to suppress the proliferation of CD4+ T cells, thus driving lung CTCs to escape from immune attacks. TGF-β can induce miR-183 to down-regulate MICA and MICB expression, which can help cancer cells evade immune detection. TGF-β1/Smad2 can up-regulate PD-L1. PD-L1 can bind to PD-1 to suppress anti-tumor immunity. TGF-β/Smad3 can induce dedicator of cytokinesis 4 (DOCK4) expression to activate Rac1. Then, Rac1 activation can promote cancer cell extravasation. TGF-β/Smad2/3 is required for the combination of histone deacetylase 4 (HDAC4), paired-like homeodomain 1 (PITX1), and roundabout, axon guidance receptor, and homolog 1 (ROBO1), which can promote the bone colonization of lung cancer cells. TGF-β can increase transcription factor 4 (TCF4), protein kinase D 3 (PRKD3), and SUSD5 levels, which can also enhance bone colonization.

Lung CTCs can produce the immune suppressors interleukin-10 (IL-10) and TGF-β. Moreover, TGF-β can increase circulating CD4+CD25+ regulatory T cells (Tregs) via the cyclooxygenase-2 (Cox-2)/prostaglandin E2 (PGE2) signaling pathway. These Tregs can suppress the proliferation of CD4+ T cells, thus driving CTCs to escape from immune attacks (Figure 2) [49]. In A549 cells, the TGF-β1/Smad2 signaling pathway can up-regulate programmed cell death ligand-1 (PD-L1) [50]. PD-L1 can bind to programmed cell death protein-1 (PD-1), expressed on activated T cells and other immune cells, ultimately suppressing anti-tumor immunity in NSCLC [51]. In H1355 and H1299 cells, TGF-β can induce miR-183 to down-regulate major histocompatibility complex class I chain-related A (MICA) and MICB expression, which can help H1355 and H1299 cells evade immune detection mediated by the circulating immune cells that express NKG2D (the transmembrane glycoproteins that can recognize MICA and MICB), thereby driving immune evasion (Figure 2) [52].

CTCs are usually arrested in narrow capillaries, where they can be trapped because of the size restriction and then extravasate [53]. In A549 cells, the TGF-β/Smad3 signaling pathway can induce a DOCK4 expression to activate Rac1. Then, Rac1 activation can extend protrusions to promote the extravasation of A549 cells (Figure 2) into distant organ sites [54]. Lung cancer cells are most likely to form metastases in the brain, bones, and lymphatic nodes [55]. In H460 cells, TGF-β can increase the levels of TCF4, PRKD3, and SUSD5. The combination of these triple factors can enhance osteolytic lesions that can increase bone colonization [56]. In A549 cells, TGF-β/Smad2/3 signaling is required for the combination of HDAC4, PITX1 and ROBO1. This combination can promote osteolytic lesions, thereby promoting bone colonization (Figure 2) of A549 cells [57].

## 4. Roles of miRNAs in TGF-β Signaling-Regulated Lung Cancer Metastasis

### 4.1. Positive Roles of miRNAs

#### 4.1.1. MiR-93

MiR-93 is up-regulated in NSCLC tissues [58]. The neural precursor cell expressed developmentally downregulated gene 4-like (NEDD4L) was predicted to be its target by miRDB and TargetScan. MiR-93 can bind to the mRNA 3′UTR of the NEDD4L and then down-regulate its expression. Down-regulated NEDD4L can increase Smad2 phosphorylation to enhance TGF-β signal transduction, finally promoting TGF-β-induced EMT in A549 and H1650 cells [59]. In addition, increased miR-93 can predict poor outcomes among NSCLC patients [60].

#### 4.1.2. MiR-128-3p

MiR-128-3p is highly expressed in NSCLC tissues compared to adjacent normal tissues. It can inhibit SMURF2 and the catalytic subunit of protein phosphatase 1 (cPP1) expressions by targeting their mRNA 3′UTRs, eventually activating the TGF-β signaling pathways to promote EMT, metastasis, and chemoresistance. Further, high miR-128-3p expression can indicate poor NSCLC patient survival [61].

#### 4.1.3. MiR-9

MiR-9 is up-regulated in NSCLC cell lines and can be up-regulated by TGF-β1. MiR-9 can promote TGF-β-induced EMT by inhibiting E-cadherin in A549 cells [62]. In addition, miR-9 can also repress SRY-Box 7 (SOX7) by targeting its mRNA 3′UTR to promote TGF-β1-mediated invasion and adhesion to fibronectin in A549 and HCC827 cells [63].

#### 4.1.4. MiR-134 and miR-487b

MiR-134 and miR-487b are both located on chromosome 14q32, and both of them can be activated by TGF-β1 during EMT progression in lung adenocarcinoma. Moreover, over-expressed miR-134 and miR-487b can promote TGF-β1-induced EMT by suppressing membrane-associated guanylate kinase, WW and PDZ domain-containing protein 2 (MAGI2) (a multidomain scaffolding protein that can repress Smad3-induced transcriptional activity by interacting with Smad3 [64]) in A549 cells [65].

#### 4.1.5. MiR-330-3p

MiR-330-3p is up-regulated in NSCLC tissues [66]. In NCI-H1975 cells, miR-330-3p expression is very high, and miR-330-3p inhibitor transfection can obviously decrease its levels. Down-regulated miR-330-3p can increase E-cadherin and reduce vimentin, thus inhibiting TGF-β1-induced EMT. This indicates a positive relationship between miR-330-3p and TGF-β1-induced EMT [67].

#### 4.1.6. MiR-1246

MiR-1246 expression has been shown to be markedly increased in NSCLC and SCLC patients’ serum by quantitative real-time polymerase chain reaction (qRT-PCR). It can decrease E-cadherin, as well as increase vimentin and TGF-β, contributing to promoting EMT in A549 cells [68].

#### 4.1.7. MiR-9-5p

MiR-9-5p levels are higher in NSCLC tissues and cell lines compared to normal ones. It has been revealed that decreased receptor effects can abrogate TGF-β suppressive functions, thereby showing its oncogenic roles in NSCLC [69,70]. MiR-9-5p can bind to TGFBR2 mRNA and then attenuate its expression, subsequently facilitating A549 cells invasion and migration [71].

#### 4.1.8. MiR-181b-5p

Cancer stem cells (CSCs) possess self-renewal properties and have been demonstrated to be a small population of cells within lung cancer tissues, which can promote lung tumor metastasis [72]. MiR-181b-5p can be obviously up-regulated by TGF-β1 in lung CSCs and A549 cells. It can suppress E-cadherin expression, thereby promoting EMT and eventually increasing invasion and metastasis in lung CSCs and A549 cells in vitro and *in vivo*. Moreover, up-regulated miR-181b-5p can predict poor outcomes in NSCLC patients [73].

#### 4.1.9. MiR-23a

**Figure 3:**
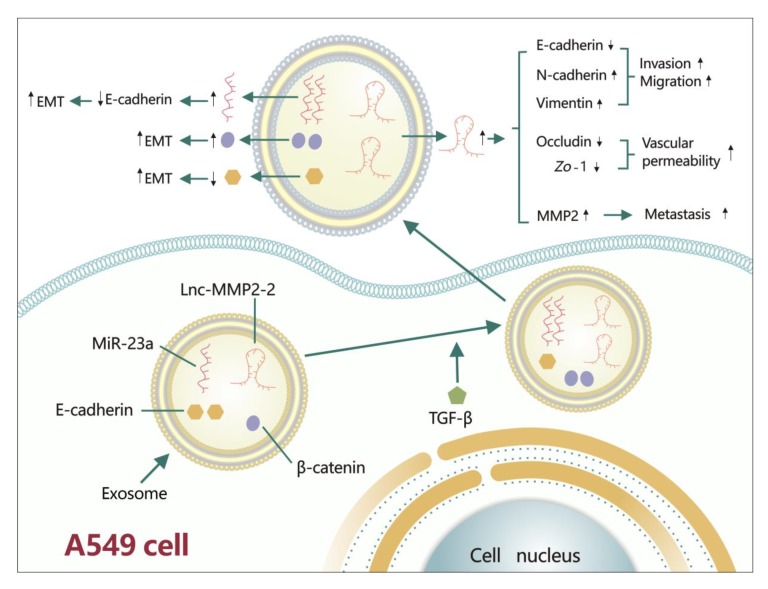
TGF-β regulates lung cancer metastasis via exosomes. TGF-β can affect lung tumor cell invasion, migration, and metastasis by modulating the cargo carried by A549 cell-derived exosomes. In this process, TGF-β can decrease E-cadherin and increase β-catenin, miR-23a and lnc-MMP2-2. Decreased E-cadherin and increased β-catenin can then promote EMT. Increased miR-23a can decrease E-cadherin to enhance EMT. Up-regulated lnc-MMP2-2 can inhibit E-cadherin, as well as increase N-cadherin and vimentin to promote invasion and migration; it can also increase vascular permeability by repressing occludin and Zo-1. Moreover, lnc-MMP2-2 can augment MMP2 to accelerate metastasis.

MiR-23a is up-regulated in NSCLC cell lines, and can also be up-regulated by the TGF-β1/Smad2/3 signaling pathway. MiR-23a can target the 3’ UTR of *CDH1* (a gene that can encode E-cadherin) and then suppress E-cadherin expression, eventually enhancing TGF-β1-induced EMT in A549 cells. Further, miR-23a can also cause resistance to gefitinib [74]. Accumulating studies have revealed that lung cancer cells can secrete exosomes into the extracellular environment to affect lung tumor cell invasion, migration, and metastasis. In this process, exosomes can carry several materials, such as DNA, nc-RNAs, and proteins [75]. MiR-23a and β-catenin can be increased by TGF-β1 in A549 cells-derived exosomes, while E-cadherin is decreased (Figure 3). In addition, treatment with these exosomes can induce EMT in A549 cells [76].

### 4.2. Negative Roles of miRNAs

#### 4.2.1. MiR-132

In NSCLC tissues with lymphatic metastasis, the expression of miR-132 is found to be obviously lower than that in adjacent normal tissues. MiR-132 can suppress the TGF-β1/Smad2 signaling pathway and, moreover, reduce N-cadherin, ZEB1, Snail, and vimentin, leading to a repression of EMT, invasion, and migration in A549 cells [77].

#### 4.2.2. MiR-203 and miR-145

MiR-203 and miR-145 are reduced in NSCLC tissues and cell lines. Smad3 was predicted to be their target by PicTar and miRanda. Both miR-203 and miR-145 can interact with the 3′UTR of Smad3 mRNA to decrease its levels, ultimately inhibiting TGF-β1-induced EMT and invasion in A549 and 95C cells [78].

#### 4.2.3. MiR-205

MiR-205 is down-regulated in NSCLC cell lines. MiR-205 can repress Smad4 by targeting its mRNA, subsequently impairing TGF-β/Smad4 signaling pathway-induced EMT in A549 cells [79]. In addition, the transfection of miR-205 mimics into NCI-H1975 cells can significantly up-regulate its expressions. Over-expressed miR-205 can reduce vimentin and increase E-cadherin to suppress TGF-β-induced EMT [67].

#### 4.2.4. MiR-124

MiR-124 is decreased in NSCLC cell lines and tissues. TGF-β can increase DNMT3a expression, and then DNMT3a can bind to a miR-124 promoter, ultimately reducing miR-124 levels. MiR-124 can interact with Smad4 mRNA 3′UTR to repress Smad4, ultimately inhibiting TGF-β-induced EMT [80].

#### 4.2.5. MiR-422a

MiR-422a is significantly down-regulated in NSCLC tissues compared to adjacent noncancerous lung tissues. MiR-422a can reduce sulfatase 2 (SULF2), which can inhibit the TGF-β1/Smad2/3 signaling pathway, eventually reducing EMT [17]. Moreover, decreased miR-422a can also be regarded as a potential biomarker of lung cancer with lymphatic metastasis [81].

#### 4.2.6. MiR-196b

MiR-196b is diminished in NSCLC cell lines compared to normal cell lines. It can attenuate TGF-β1-induced EMT by decreasing N-cadherin, vimentin, ZEB1, and Snail in A549 cells [82].

#### 4.2.7. MiR-940

MiR-940 is declined in NSCLC tissues. It can target Snail mRNA 3′UTR and repress Snail expression, thereby inhibiting TGF-β1-induced EMT in A549 and H226 cells [83].

#### 4.2.8. MiR-22

MiR-22 is obviously down-regulated in NSCLC cell lines. MiR-22 can be decreased by TGF-β1 in Anip973 and AGZY83-a cells. It can reduce Snail expression by binding to Snail mRNA 3′UTR, thereby suppressing TGF-β1-induced EMT [84].

#### 4.2.9. MiR-200 Family

The miR-200 family consists of five members, including miR-200b, miR-200a, miR-429, miR-200c, and miR-141. The genes *miR-200a* and *miR-200c* can be repressed by TGF-β through over-expressing JARID2 and embryonic ectoderm development (EED) during EMT progression. *miR-200a* and *miR-200c* can inhibit ZEB1 and ZEB2 expression to repress EMT in A549 cells [85,86].

#### 4.2.10. MiR-145 and miR-497

MiR-145 and miR-497 levels are decreased in NSCLC cell lines compared to normal cell lines. MiR-145 and miR-497 can inhibit metadherin (MTDH) by targeting its mRNA 3′UTR, thereby inhibiting TGF-β-induced EMT, migration, and invasion in A549 and H1299 cells [87].

#### 4.2.11. MiR-149

The expression of miR-149 is higher in low-invasive NSCLC cells (A549 and Calu3 cells) than in high-invasive NSCLC cells (H1299 cells). MiR-149 can target forkhead Box M1 (FOXM1) mRNA 3’ UTR and repress its expression, eventually leading to a suppression of TGF-β1-induced EMT in A549 cells [88].

#### 4.2.12. MiR-134

MiR-134 expression is also higher in A549 and Calu1 cells than that in H1299 cells, and miR-134 can also repress forkhead Box M1 (FOXM1) to abrogate TGF-β1-induced EMT in A549 cells [89].

#### 4.2.13. MiR-133

MiR-133 is decreased in NSCLC cell lines. In A549 cells, miR-133 can down-regulate forkhead box Q1 (FOXQ1) to reduce TGF-β expression, finally suppressing EMT [90].

#### 4.2.14. MiR-29c

MiR-29c is down-regulated in NSCLC cell lines compared to normal cell lines. MiR-29c can be reduced by TGF-β1. MiR-29c can inhibit specificity protein 1 (Sp1), thereby repressing TGF-β1-induced EMT in A549 and 95C cells [91].

#### 4.2.15. MiR-16

MiR-16 is decreased in human NSCLC tissues compared to adjacent normal tissues. Autophagy is a complex biological process associated with the lysosomal degradation of cellular proteins and other cytoplasmic contents; it can also suppress NSCLC metastasis [92,93]. MiR-16 mimics were transfected into A549 cells to increase their expression levels. Up-regulated miR-16 can activate autophagy to inhibit TGF-β1-induced EMT [94].

#### 4.2.16. MiR-129

MiR-129 is down-regulated in NSCLC tissues [95]. Overexpressed miR-129 can inhibit sex-determining region Y-box 4 (SOX4) expression, ultimately leading to abrogating TGF-β-induced EMT in A549 cells [96]. Decreased miR-129 can predict a poor prognosis of NSCLC patients [95].

#### 4.2.17. MiR-485-5p and miR-3127-5p

MiR-485-5p and miR-3127-5p are both significantly lower in NSCLC tissues than in adjacent normal lung tissues. Both of them can impair TGF-β-induced EMT by preventing vimentin induction and increasing E-cadherin in A549 cells [97,98].

#### 4.2.18. MiR-3607-3p

MiR-3607-3p expression is lower in NSCLC tissues than that in adjacent normal lung tissues. It can bind to the 3′UTR of both TGFBR1 and cyclin E2 (CCNE2) mRNAs and then repress their expressions, thereby inhibiting NSCLC invasion and migration [99].

#### 4.2.19. MiR-206 and miR-140

MiR-206 and miR-140 are decreased in NSCLC tissues. Both miR-206 and miR-140 can interact with 3′UTR of Smad3 mRNA to down-regulate its expression, thereby suppressing the TGF-β1 signaling pathway and ultimately abrogating A549 cell invasion, migration, and metastasis [100].

#### 4.2.20. MiR-136

MiR-136 has been found to be over-expressed in NSCLC tissues compared to adjacent normal lung tissues [101]. In A549 and Calu-3 cells, miR-136 can reduce cell invasion and migration by reducing Smad2 and Smad3 expression, which may be involved in attenuating the TGF-β signaling pathway [102].

#### 4.2.21. MiR-133a

MiR-133a is also down-regulated in lung adenocarcinoma cells. MiR-133a can abrogate AKT signaling by reducing the TGF-β/TGFBR1 signal, contributing to suppressing A549 cell invasion and migration. In addition, low miR-133a expression can indicate a poor overall survival for lung cancer patients [103].

#### 4.2.22. MiR-143

MiR-143 is obviously reduced in NSCLC tissues compared to normal lung tissues. It can be stimulated by the TGF-β/Smads signaling pathway. MiR-143 can inhibit Smad3, CD44, and K-Ras expression, eventually repressing cell invasion and migration in A549 cells [104].

#### 4.2.23. MiR-886-3p

The miR-886-3p level is low in SCLC tissues and cell lines. MiR-886-3p can reduce cell invasion and migration by repressing TGF-β1 in NCI-H446 cells. In addition, down-regulated miR-886-3p can predict a shorter survival for SCLC patients [105].

#### 4.2.24. MiR-138

MiR-138 can be down-regulated by TGF-β1 in LC006 and LC021 cells. It can inhibit CSC formation by suppressing TGF-β1-induced EMT in primary lung cancer cells [106].

## 5. Roles of lncRNAs in TGF-β Signaling-Regulated Lung Cancer Metastasis

### 5.1. Positive Roles of lncRNAs

#### 5.1.1. LncRNA HCP5

LncRNA histocompatibility leukocyte antigen complex P5 (HCP5) is highly expressed in lung adenocarcinoma cell lines, especially in current smokers and patients with mutated *EGFR* and *KRAS*. HCP5 can be induced by the TGF-β/Smad3 signaling pathway. In addition, it can augment Snail and Slug expression by attenuating miR-203, which can promote TGF-β/Smad3 signaling-induced EMT and invasion. Furthermore, HCP5 over-expression can indicate a poor prognosis for lung adenocarcinoma patients [107].

#### 5.1.2. LncRNA NORAD

LncRNA NORAD expression is higher in NSCLC tissues than in adjacent normal tissues [108]. NORAD can stimulate Smad3 and importin β1 complex formation and then promote the TGF-β-induced translocation of the Smad2/3 complexes into the cell nucleus. NORAD ultimately increases EMT-related gene (*SNAI1* and *FN1,* which can encode Snail and fibronectin, respectively) expression to enhance TGF-β-induced EMT [18].

#### 5.1.3. LncRNA XIST

LncRNA X inactivate-specific transcript (XIST) is up-regulated in NSCLC tissues. On the one hand, XIST can bind to miR-137 and decrease its expression, thereby suppressing miR-137′s inhibitory roles in TGF-β1-induced EMT [20]. On the other hand, it can act as a kind of miRNA sponge to reduce miR-367 and miR-141 expression, thereby increasing ZEB2 [19]. Through these two mechanisms, XIST can facilitate TGF-β1-induced EMT in A549 cells [19,20].

#### 5.1.4. LncRNA linc00673

LncRNA linc00673 is up-regulated in NSCLC tissues and cell lines. It can also be elevated by TGF-β. MiR-150-5p can target ZEB1 mRNA 3′UTR and attenuate its expression. Linc00673 can act as a competing endogenous RNA (ceRNA) that can suppress the effect of miR-150-5p on ZEB1, ultimately potentiating TGF-β-induced EMT, invasion, and migration in A549 and H1975 cells. Furthermore, up-regulated linc00673 can indicate poor survival for NSCLC patients [109].

#### 5.1.5. LncRNA MEG3

LncRNA MEG3 can be overexpressed by TGF-β during EMT progression. In A549 and LC-2/ad cells, MEG3 shRNA can silence its expression, and MEG3 knockdown can abrogate TGF-β-induced actin stress fiber formation, which can inhibit EMT. MEG3 cDNA can increase its levels, and MEG3 over-expression can not only decrease the expression of genes *CDH1*, *miR-200a,* and *miR-200c* but can also increase the expression of genes *ZEB1* and *ZEB2* through the recruitment of JARID2 and the enhancer of zeste homologue 2 (EZH2) [110].

#### 5.1.6. LncRNA MEG8

LncRNA MEG8 can also be up-regulated by TGF-β during EMT progression. MEG8 can interact with EZH2 and can subsequently down-regulate *CDH1* expression. At the same time, MEG8 can up-regulate *SNAI1* and *SNAI2* (a gene that can encode Slug) expression by repressing the *miR-34a* and *miR-203* genes in A549 and LC-2/ad cells. It has also been revealed that neither MEG8 nor MEG3 can exclusively lead to EMT without TGF-β. However, the co-expression of MEG8 and MEG3 can induce EMT in the absence of TGF-β [111].

#### 5.1.7. LncRNA ATB

LncRNA activated by TGF-β (ATB) is obviously over-expressed in NSCLC tissues. ATB can accelerate NSCLC migration by abolishing E-cadherin and increasing N-cadherin [112]. Additionally, ATB can enhance TGF-β1-induced EMT by down-regulating miR-494 [113]. ATB can also inhibit miR-494 to activate the AKT and the JAK/STAT3 pathways that are required for TGF-β-induced EMT [38]. Notably, high lncRNA-ATB expression can predict poor outcomes for lung cancer patients [114].

#### 5.1.8. LncRNA TBILA

LncRNA TGF-β-induced lncRNA (TBILA) is highly expressed in NSCLC tissues and can be induced by the TGF-β1/Smad2/3 signaling pathway. TBILA can not only activate RhoA by up-regulating human germinal center-associated lymphoma (HGAL) (a TGF-β-induced gene) but can also increase S100A7/c-Jun activation domain-binding protein 1 (JAB1) activity by interacting with S100A7, eventually facilitating invasion and migration in A549 cells [115].

#### 5.1.9. Lnc-MMP2-2

Lnc-MMP2-2 is over-expressed in lung cancer tissues, and it can be obviously increased in the exosomes derived from A549 cells by TGF-β (Figure 3). On the one hand, exosomal lnc-MMP2-2 can increase N-cadherin and vimentin and decrease E-cadherin to facilitate invasion and migration. On the other hand, it can inhibit occludin and zonula occludens-1 (Zo-1) to impair vascular tight junctions, thus increasing vascular permeability. Exosomal lnc-MMP2-2 can also increase MMP2, which can accelerate metastasis [116].

#### 5.1.10. LncRNA AWPPH

LncRNA AWPPH expression was estimated to be higher in the blood of lung cancer patients than in normal people by qRT-PCR. AWPPH can stimulate TGF-β1 protein expression to promote NSCLC migration and invasion. Moreover, high AWPPH can indicate the postoperative distant recurrence of NSCLC patients [117].

#### 5.1.11. LncRNA CASC11

LncRNA CASC11 has been shown by qRT-PCR to be significantly increased in the plasma of SCLC patients. CASC11 can promote SCLC stemness by up-regulating TGF-β1, eventually increasing the number of lung CSCs. Further, CASC11 up-regulation can reveal the poor prognosis of SCLC patients [118].

### 5.2. Negative Roles of lncRNAs

#### 5.2.1. LncRNA HAND2-AS1

LncRNA HAND2-AS1 is down-regulated in NSCLC tissues. HAND2-AS1 can inhibit migration, invasion, and stemness by reducing TGF-β1 expression, as well as phosphorylated Smad2 and Smad3 levels, in NSCLC [119].

#### 5.2.2. LncRNA linc01186

LncRNA linc01186 is decreased in NSCLC tissues. It can be repressed by the TGF-β1/Smad3 signaling pathway. Linc01186 can inhibit cell invasion and migration by repressing EMT [120].

#### 5.2.3. LncRNA LINP1

LncRNA in nonhomologous end joining (NHEJ) pathway 1 (LINP1) is also reduced by TGF-β1/Smad4. LINP1 can increase E-cadherin and downregulate vimentin and Snail to abrogate TGF-β1-induced EMT and cell invasion [121].

#### 5.2.4. LncRNA ANCR

LncRNA ANCR has been shown by qRT-PCR to be markedly decreased in the plasma of NSCLC patients. Moreover, it can decrease TGF-β1 expression and thus inhibit NSCLC migration and invasion. Low ANCR expression can reveal the shorter overall survival time of patients than that of patients with high ANCR expression [122].

#### 5.2.5. LncRNA NKILA

LncRNA NF-κB Interacting LncRNA (NKILA) is down-regulated in NSCLC tissues. NKILA can be increased by TGF-β/Smads pathways. It can suppress invasion and migration by inhibiting NF-κB activation and then repressing Snail-mediated EMT [123].

## 6. MiRNA Sponges Participate in TGF-β Signaling-Regulated Lung Cancer Metastasis

LncRNAs, ceRNAs, and circular RNAs (circRNAs) have been regarded as miRNA sponges to inhibit miRNAs in lung cancer. LncRNA has MREs, and it can influence the target gene downstream of the miRNAs by repressing them [124]. CeRNAs can regulate gene expression by competitively binding to miRNA through MREs, thereby suppressing the interactions between miRNA and its targeted mRNA. CeRNA is also essential for miRNA and lncRNA regulatory networks [125]. CircRNAs, belonging to endogenous nc-RNAs, possess closed-loop structures without a 5’ end cap and a 3’ end poly tail structure. CircRNA has several miRNA-binding sites to adsorb miRNA, subsequently counteracting miRNA’s effects on its target gene expression [126]. Additionally, circRNAs can also work as ceRNAs in lung cancer. CeRNAs and circRNAs can regulate lung cancer migration, invasion, and metastasis [127,128].

### 6.1. CeRNA

#### 6.1.1. CeRNA MYEOV

The ceRNA myeloma overexpressed gene (MYEOV) is significantly over-expressed in NSCLC tissues compared to adjacent normal tissues. MiR-30c-2-3p can inhibit TGFBR2 and Ubiquitin specific protease 15 (USP15) (a deubiquitinating enzyme that can promote TGF-β signaling) expression by targeting their mRNA 3′UTRs. MYEOV can bind to and then suppress miR-30c-2-3p, eventually contributing to activating TGF-β signaling and potentiating invasion and metastasis in NSCLC [129].

#### 6.1.2. CeRNA HMGA2

CeRNA HMGA2 is up-regulated in metastatic lung adenocarcinoma. HMGA2 can increase TGFBR3 expression by repressing miRNA let-7, which can also enhance the TGF-β signaling pathway, ultimately promoting invasion and metastasis in NSCLC [130].

### 6.2. CircRNA

The circRNA CircPTK2 level is lower in NSCLC tissues than in normal lung tissues. It can be inhibited by TGF-β1 during EMT progression. MiR-429 and miR-200b-3p can repress transcriptional intermediary factor 1 γ (TIF1γ) (a regulator that can suppress TGF-β/Smad signaling [131]) expression. CircPTK2 can bind to miR-429 and miR-200b-3p, subsequently attenuating their effects and resulting in abrogating TGF-β1-induced EMT, migration, and invasion in A549 cells [132].

## 7. Targeted Therapy

Although traditional lung cancer therapies, such as surgical resection, chemotherapy, and radiotherapy, have made great progress, they still face some limitations. For example, drug resistance and lung cancer distant metastasis can usually influence the therapeutic effects of a treatment. As a result, it is necessary to develop new strategies. MiRNAs and lncRNAs participate in substantial lung cancer progression, and we may be able to use them as treatment targets.

### 7.1. Curcumin

Curcumin, isolated from *Curcuma longa*, possesses anti-cancer effects and chemopreventive properties. Curcumin can significantly inhibit several miRNA-associated pathways, such as the MAPK, TGF-β, and Wnt signaling pathways, contributing to suppressing invasion and migration in A549 cells [133].

### 7.2. Carboplatin

Carboplatin can interact with ifosfamide and paclitaxel and possesses fewer non-hematologic toxicities than cisplatin (DDP). Carboplatin is a chemotherapeutic drug widely used to treat SCLC and NSCLC in order to improve overall survival [134]. Carboplatin can suppress miR-21 to increase Smad7 expression. Then, up-regulated Smad7 can inhibit the TGF-β/Smads signaling pathways, thereby suppressing TGF-β-mediated invasion in A549 cells [135].

### 7.3. DDP

DDP is also extensively used to deal with lung cancer. MiR-181b is decreased in NSCLC cells. It can target TGFBR1 mRNA 3′UTR and repress its expression, thus abrogating the TGF-β signal and eventually increasing DDP chemosensitivity in NSCLC cells [136]. MiR-17, miR-20a, and miR-20b are all down-regulated in A549/DDP cells (DDP-resistant A549 cells). They can attenuate the TGF-β signal pathway by inhibiting TGFBR1, thereby reducing migration and increasing DDP sensitivity in A549/DDP cells [137].

### 7.4. DAC

Decitabine (DAC) is a deoxyribonucleotide and can abrogate DNA methylation. Functionally, DAC promotes demethylation of the miR-200 promoter to increase the levels of miR-200c and miR-200a, which can repress *ZEB1* and *ZEB2* expression, leading to decreasing TGF-β1-induced EMT and metastasis in PC9 cells [138].

## 8. Discussion and Conclusions

Lung cancer is the most common cancer in men and the second most common in women after breast cancer. The low overall survival rate of lung cancer patients is usually correlated with distant organ metastasis. MiRNAs and lncRNAs are demonstrated to be oncogenes or suppressors by targeting mRNA 3′UTR in lung cancer development. Currently, miRNAs and lncRNAs are widely used to explore lung cancer’s metastatic mechanisms, and these two nc-RNAs are increasingly used as biomarkers to predict the outcomes of lung cancer patients. TGF-β is involved in many cancers, and TGF-β signaling pathways have also been extensively confirmed to play pivotal roles in lung cancer metastasis. Interestingly, we found that Carboplatin, the first-line therapy used to treat metastatic lung cancer, suppresses TGF-β signaling-regulated lung cancer metastasis by repressing miR-21 [135]. This result suggests a potential treatment strategy involving miRNAs and TGF-β signaling for lung cancer metastasis. Considering the widespread attention attracted by the rational design of miRNA/lncRNA-targeted drugs against lung cancer, we have summarized and discussed the relationships between these two nc-RNAs and TGF-β signaling pathways in lung cancer metastasis. We hope that this review will provide new ideas for more clinical therapies like Carboplatin, thereby allowing us to treat lung cancer more effectively.

In the current review, we discussed how miRNAs and lncRNAs can exert positive (Table 1) and negative (Table 2) influences on TGF-β signaling-regulated lung cancer metastasis. The positive roles of miRNAs and lncRNAs can be concluded as follows: miR-93 [59], miR-128-3p [61], miR-9 [62], miR-134, miR-487b [65], miR-330-3p [67], miR-1246 [68], miR-23a [74], HCP5 [107], NORAD [18], XIST [19,20], linc00673 [109], MEG3 [110], MEG8 [111], and ATB [113] can enhance TGF-β signaling-induced EMT. MiR-9 [63], miR-9-5p [71], ATB [112], TBILA [115] lnc-MMP2-2 [116], and AWPPH [117] can accelerate TGF-β signaling-mediated lung cancer cell migration and invasion. Lnc-MMP2-2 can also increase vascular permeability [116]. TGF-β1-increased miR-181b-5p can facilitate lung CSCs’ metastatic abilities by inducing EMT [73]. CASC11 can augment TGF-β1 to promote lung cancer cell stemness [139]. TGF-β-induced miR-183 can down-regulate MICA and MICB to make NSCLC cells escape from immune attacks mediated by circulating immune cells (i.e., natural killer (NK) cells and CD8+ T cells) expressing NKG2D [52]. Additionally, in the NSCLC microenvironment, tumor-derived TGF-β can also increase miR-183, which can decrease DNAX activating protein 12 kDa (DAP12) (a protein required for NK cell cytotoxicity functions), ultimately inhibiting tumor-infiltrating NK cell activities to promote immunosuppression [140], while miRNAs and lncRNAs can also exhibit opposite effects on lung cancer metastasis. MiR-132 [77], miR-203, miR-145 [78], miR-205 [79], miR-124 [80], miR-422a [17], miR-196b [82], miR-940 [83], miR-22 [84], *miR-200a*, *miR-200c* [85,86], miR-145, miR-497 [87], miR-149 [88], miR-134 [89], miR-133 [90], miR-29c [91], miR-16 [94], miR-129 [96], miR-485-5p [97], miR-3127-5p [98], and LINP1 [121] can suppress TGF-β signaling-regulated EMT. MiR-3607-3p [99], miR-206, miR-140 [100], miR-136 [102], miR-133a [103], miR-143 [104], miR-886-3p [105], HAND2-AS1 [119], linc01186 [120], ANCR [122], and NKILA [123] can repress TGF-β signaling-modulated lung cancer cell migration and invasion. MiR-138 can reduce lung CSC formation by abrogating TGF-β1-induced EMT [106]. HAND2-AS1 can attenuate lung cancer cell stemness by inhibiting the TGF-β1/Smad2/3 signaling pathway [119]. During these progressions, we found the following similarities between miRNAs and lncRNAs (Table 1, Table 2, and Figure 3):

They can be increased or decreased via TGF-β signaling. MiR-9 [62,63], miR-23a [74,76], miR-134, miR-487b [65], miR-181b-5p [73], miR-143 [104], HCP5 [107], linc00673 [109], MEG3 [110], MEG8 [111], ATB [113], TBILA [115], lnc-MMP2-2 [116], and NKILA [123] can be increased by TGF-β signaling, while *miR-200a*, *miR-200c* [85,86], miR-138 [106], linc01186 [120], and LINP1 [121] can be decreased by TGF-β signaling.

➣ They can promote or suppress TGF-β/Smads pathways or other non-Smad pathways. MiR-93 [59] and NORAD [18] can promote the TGF-β/Smads signaling pathways. ATB can promote the AKT signal and JAK/STAT3 signal [113]. MiR-132 [77], miR-422a [17], miR-206, miR-140 [100], and HAND2-AS1 [119] can repress the TGF-β/Smads signaling pathways. MiR-133a [103] and NKILA [123] can repress the TGF-β/AKT and NF-κB/Snail pathway, respectively.
➣ They can promote or inhibit lung CSC formation. CASC11 can promote lung CSC formation [118], while miR-138 [106] and HAND2-AS1 [119] can inhibit lung CSC formation.
➣ They can be up-regulated in TGF-β-mediated exosomes, including miR-23a [76] and lnc-MMP2-2 [116].

MiRNAs and lncRNAs can be detected in biological fluids through a biopsy of liquids, such as whole blood (lncRNA metastasis-associated lung adenocarcinoma transcript 1 (MALAT1) [141] and miRNA-182-5p [142]), serum (lncRNA linc00342 [143], MEG3 [144], miR-96 [145] and miR-30a-5p [146]), plasma (lncRNA linc00152 [147], MALAT1 [148], miR-145 [149], miR-30b [150], miR-25 [151] and miR-425-3p [152]), sputum (lncRNA HOTAIR, H19 [153], miR-21 and miR-210 [154]), and pleural effusion (MALAT1 [155] and miR-200 family [156]) in lung cancer. Furthermore, miRNAs can also be found in bronchoalveolar lavage fluid (miR-126 and miR-Let-7a [157]). Both lncRNAs and miRNAs are usually dysregulated in lung cancer and are closely related to cancer metastasis. For instance, miR-25 is up-regulated in NSCLC tissues and cell lines and can repress large tumor suppressor homology 2 (LATS2) and then increase the yes-associated protein (YAP), ultimately contributing to promoting A549 cells migration and invasion [158]. Moreover, high plasma miR-25 levels can indicate a poor prognosis for NSCLC patients [151]. MiR-96 is elevated in the serum-derived exosomes of lung cancer patients. MiR-96 can suppress LIM-domain only protein 7 (LMO7) expression, thereby promoting A549 cell proliferation, migration, and DDP resistance. In addition, increasing serum exosomal miR-96 can predict lung cancer lymph node metastasis [145]. MiR-21-5p is highly expressed in NSCLC tissues. It can decrease E-cadherin and increase MMP9 and vimentin, which can facilitate A549 cell invasion and migration [159]. Further, high plasma miR-21 can reveal the distant metastasis of NSCLC patients [160]. The expression of MALAT1 is significantly high in NSCLC tissues and cell lines [161]. MALAT1 can enhance A549 cell invasion and migration by repressing miR-200a-3p [162]. High serum exosomal MALAT1 could be an indicator for lymphatic metastasis in NSCLC patients [163]. Linc00152 is up-regulated in lung adenocarcinoma tissues and can enhance H1299 and H1975 cell migration and invasion [164]. Additionally, increased plasma linc00152 levels can indicate recurrence in NSCLC patients [147]. Linc00342 is up-regulated in NSCLC tissues and cell lines. Linc00342 can increase cell invasion and migration by inhibiting miR-203a-3p in NCI-H1299 and NCI-H460 cells [165], and high serum linc00342 can act as a better biomarker to diagnose NSCLC than CYFRA 21-1 (a sensitive factor that can diagnose lung cancer squamous cell carcinoma [166]) [143]. In addition to circulating miRNAs and lncRNAs, the existence of lung cancer CTCs can also predict a poor outcome for lung cancer patients. For example, the presence of CTCs at six months after surgery can increase the risk of metastasis and the short relapse-free survival of lung cancer patients [167]. It has been shown that molecular characterizations (proteins, RNAs, etc.) are closely related to lung cancer detection, prognosis, and therapeutic efficacy [168]. Therefore, miRNA, as a type of molecular characterization, might provide new ideas for the diagnosis of lung cancer metastasis, prognostic prediction, and therapeutic selection in lung cancer patients.

Due to the multiple functions of miRNAs and lncRNAs in lung cancer metastasis, these RNAs have been regarded as promising therapeutic targets. Several traditional treatments have been discovered to target miRNAs and lncRNAs. Low-dose radiation can increase miR-30a and miR-30b expression. MiR-30a and miR-30b can inhibit plasminogen activator inhibitor-1 (PAI-1) expression and then reduce EMT and migration in NCI-H460 cells exposed to high-dose radiation. Additionally, miR-30a and miR-30b also promoted sensitivity to radiotherapy in a mouse model [169]. In Lewis lung cancer cells, radiation can attenuate HOTAIR expression to reduce the nuclear localization of β-catenin, thereby inhibiting tumor cell proliferation [170]. The effective chemotherapeutic drug, paclitaxel (PTX), can increase MEG3, thus promoting A549 cell apoptosis by increasing p53 expression. Further, over-expressed MEG3 can strengthen PTX’s anti-tumor activity in A549 cells [171]. DDP can increase miR-425-3p in cells and NSCLC cell-derived exosomes. Exosomal miR-425-3p can activate autophagy by inhibiting AKT1, ultimately repressing DDP sensitivity in A549 cells [172]. MiRNAs can also exert therapeutic effects. MiR-200c can be delivered to lung tumor cells through nano vehicles. Nano miR-200c can increase the expressions of the tumor suppressors miR-29b and miR-1247 in metastatic and non-metastatic lung cancer cells. It can also suppress lung tumor cell migration and invasion [173]. Tumor-derived exosomes can selectively deliver anticancer therapies to lung cancer sites due to their intrinsic organotropic tumor-homing properties [174,175,176]. Functionalization of exosome-mimetic nanosystems (EMNs) with ITGα6β4 (F-EMNs) have similar structures and capabilities against tumor-derived exosomes and can escape endolysosomal degradation. MiR-145 can repress N-cadherin expression and then inhibit A549 cell migration and invasion [177]. The injection of F-EMNs loaded with therapeutic oligonucleotide miR-145 can effectively reach lung tumor sites. Despite the nonnegligible safety concerns caused by their low systemic toxicities, this method remains a promising approach to target lung cancer cells during the targeted therapy process [178]. It has been indicated that nanomedicine inhalation can also be used to treat lung cancer [179]. One study found that siRNA combined with PTX inhalation via nanostructured lipid carriers can significantly decrease lung tumor volumes [180]. Copper oxide nanoparticles can deliver miR-29b to A549 cells [181], and this may inhibit lung tumor cells’ migration, invasion, and metastasis [182]. Taken together, nano-miRNA inhalation might be a new strategy to deal with lung cancer metastasis, with few potential off-target effects and systemic toxicities [183].

Presently, despite the great progress that has been made in traditional lung cancer treatments, there are still multifaceted limitations impairing these treatments’ effects, such as therapeutic resistance and drug toxicity. As mentioned above, miRNAs and lncRNAs are widely involved in TGF-β signaling-regulated lung cancer metastasis. Targeting the miRNA/lncRNA-TGF-β signaling axis might provide new insights to decrease lung cancer metastasis and improve the prognosis of lung cancer patients. However, these studies are performed mostly on NSCLC cell lines, and studies on SCLC cell lines and in lung cancer tissues are still lacking. Therefore, further explorations in these microenvironments are required to elucidate the roles of miRNAs and lncRNAs in lung cancer more completely.

## Figures and Tables

**Table 1:** The positive roles of miRNAs and lncRNAs in TGF-β signaling-regulated lung cancer metastasis.

Nc-RNA	Cell Lines	Roles	References
miR-93	A549, H1650	miR-93→NEDD4L↓→TGF-β/Smad2 signal↑→EMT↑	[59]
miR-128-3p	A549	miR-128-3p→SMURF2↓, cPP1↓→TGF-β signal↑→EMT↑, metastasis↑, chemoresistance↑	[61]
miR-9	A549	miR-9→E-cadherin↓→EMT↑	[62]
miR-23a	A549	miR-23a→E-cadherin↓→EMT↑	[74]
miR-134, miR-487b	A549	miR-134, miR-487b→MAGI2↓→EMT↑	[65]
miR-330-3p	NCI-H1975	miR-330-3p→E-cadherin↓, vimentin↑→EMT↑	[67]
miR-1246	A549	miR-1246→E-cadherin↓, vimentin↑, TGF-β↑→EMT↑	[68]
miR-9	A549, HCC827	miR-9→SOX7↓→invasion↑, adhesion↑	[63]
miR-9-5p	A549, SK-MES-1	miR-9-5p→TGFBR2↓→invasion↑, migration↑	[71]
miR-181b-5p	A549	miR-181b-5p→E-cadherin↓→EMT↑→invasion↑, metastasis↑	[73]
lncRNA HCP5	A549	HCP5→miR-203↓→Snail↑, Slug↑→EMT↑, invasion↑	[107]
lncRNA NORAD	A549	NORAD→Smad3/importin β1 complex formation↑→TGF-β/Smad2/3 signal↑→*SNAI1*↑, *FN1*↑→EMT↑	[18]
lncRNA XIST	A549	XIST→miR-137↓→EMT↑	[20]
		XIST→miR-367↓, miR-141↓→ZEB2↑→EMT↑	[19]
lncRNA linc00673	A549, H1975	linc00673→miR-150-5p↓→ZEB1↑→EMT↑, invasion↑, migration↑	[109]
lncRNA MEG3	A549, LC-2/ad	MEG3→*CDH1*↓, *miR-200a*↓, *miR-200c*↓, *ZEB1*↑, *ZEB2*↑→EMT↑	[110]
lncRNA MEG8	A549	MEG8→*miR-34a*↓, *miR-203*↓→*SNAI1*↑, *SNAI2*↑→EMT↑	[111]
lncRNA ATB	A549, HCC827	ATB→E-cadherin↓, N-cadherin↑→migration↑	[112]
	A549	ATB→miR-494↓→AKT signal↑, JAK/STAT3 signal↑→EMT↑	[113]
lncRNA TBILA	A549	TBILA→HGAL↑, RhoA↑, S100A7/ JAB1 signal↑→invasion↑, migration↑	[115]
lnc-MMP2-2	A549	lnc-MMP2-2→E-cadherin↓, N-cadherin↑, vimentin↑→invasion↑, migration↑	[116]
		lnc-MMP2-2→occludin↓, Zo-1↓→vascular permeability↑	
		lnc-MMP2-2→MMP2↑→metastasis↑	
lncRNA AWPPH	NCI-H1993, NCI-H2170	AWPPH→TGF-β1↑→invasion↑, migration↑	[117]
lncRNA CASC11	SHP-77, DMS79	CASC11→TGF-β1↑→stemness↑	[118]

**Abbreviations:** nc-RNA, non-coding RNA; miR, microRNA; NEDD4L, Neural precursor cell expressed developmentally downregulated gene 4-like; TGF-β, transforming growth factor-β; EMT, epithelial-mesenchymal transition; SMURF2, Smad ubiquitin regulatory factor 2; cPP1, catalytic subunit of protein phosphatase 1; MAGI2, membrane-associated guanylate kinase, WW and PDZ domain-containing protein 2; SOX7, SRY-Box 7; TGFBR2, TGF-β receptor 2; lncRNA, long non-coding RNA; HCP5, histocompatibility leukocyte antigen complex P5; XIST, X inactivate-specific transcript; ZEB2, Zinc finger E-box-binding homeobox 2; lncRNA-ATB, lncRNA activated by TGF-β; AKT, protein kinase B; JAK, Janus-activated kinase; STAT3, signal transducer and activator of transcription-3; TBILA, TGF-β-induced lncRNA; HGAL, human germinal center-associated lymphoma; JAB1, c-Jun activation domain-binding protein 1; Zo-1, zonula occludens-1; MMP2, matrix metalloprotease 2.

**Table 2:** The negative roles of miRNAs and lncRNAs in TGF-β signaling-regulated lung cancer metastasis.

Nc-RNA	Cell Lines	Roles	References
miR-132	A549	miR-132→TGF-β1/Smad2 signal↓→EMT↓, invasion↓, migration↓	[77]
miR-203, miR-145	A549, 95C	miR-203, miR-145→Smad3↓→EMT↓, invasion↓	[78]
miR-124	A549	miR-124→Smad4↓→EMT↓	[80]
miR-205	NCI-H1975	miR-205→E-cadherin↑, vimentin↓→EMT↓	[67]
	A549	miR-205→Smad4↓→EMT↓	[79]
miR-422a	H522	miR-422a→SULF2↓→TGF-β1/Smad2/3 signal↓→EMT↓	[17]
miR-196b	A549	miR-196b→N-cadherin↓, vimentin↓, ZEB1↓, Snail↓→EMT↓	[82]
miR-940	A549, H226	miR-940→Snail↓→EMT↓	[83]
miR-22	Anip973, AGZY83-a	miR-22→Snail↓→EMT↓	[84]
*miR-200a*, *miR-200c*	A549	*miR-200a*, *miR-200c*→ZEB1↓, ZEB2↓→EMT↓	[85,86]
miR-145, miR-497	A549, H1299	miR-145, miR-497→MTDH↓→EMT↓,invasion↓, migration↓	[87]
miR-149	A549	miR-149→FOXM1↓→EMT↓	[88]
miR-134	A549	miR-134→FOXM1↓→EMT↓	[89]
miR-133	A549, HCC827	miR-133→FOXQ1↓→TGF-β↓→EMT↓	[90]
miR-29c	95C, A549	miR-29c→Sp1↓→EMT↓	[91]
miR-16	A549	miR-16→autophagy↑→EMT↓	[94]
miR-129	A549	miR-129→SOX4↓→EMT↓	[96]
miR-485-5p	A549	miR-485-5p→E-cadherin↑, vimentin↓→EMT↓	[97]
miR-3127-5p	A549, H1299	miR-3127-5p→E-cadherin↑, vimentin↓→EMT↓	[98]
miR-3607-3p	H157, H292	miR-3607-3p→TGFBR1↓, CCNE2↓→ invasion↓, migration↓	[99]
miR-206, miR-140	A549	miR-206, miR-140→TGF-β1/Smad3 signal↓→invasion↓, migration↓, metastasis↓	[100]
miR-136	A549, Calu-3	miR-136→Smad2↓, Smad3↓→invasion↓, migration↓	[102]
miR-133a	A549	miR-133a→TGF-β/TGFBR1 signal↓→AKT signal↓→invasion↓, migration↓	[103]
miR-143	A549	miR-143→Smad3↓, CD44↓, K-Ras↓→invasion↓, migration↓	[104]
miR-886-3p	NCI-H446	miR-886-3p→TGF-β1↓→invasion↓, migration↓	[105]
miR-138	LC006, LC021	miR-138→EMT↓→CSCs formation↓	[106]
lncRNA HAND2-AS1	H1581, H1993	HAND2-AS1→TGF-β1/Smad2/3 signal↓→invasion↓, migration↓, stemness↓	[119]
lncRNA linc01186	A549	linc01186→EMT↓→invasion↓, migration↓	[120]
lncRNA LINP1	A549	LINP1→E-cadherin↑, vimentin↓, Snail↓→EMT↓	[121]
lncRNA ANCR	NCI-H23, NCI-H522	ANCR→TGF-β1↓→invasion↓, migration↓	[122]
lncRNA NKILA	A549, H226	NKILA→NF-κB↓→Snail↓→EMT↓→invasion↓, migration↓	[123]

**Abbreviations:** nc-RNA, non-coding RNA; miR, microRNA; EMT, epithelial-mesenchymal transition; TGF-β, transforming growth factor-β; SULF2, Sulfatase 2; ZEB1, Zinc finger E-box-binding homeobox 1; MTDH, metadherin; FOXM1, forkhead Box M1; FOXQ1, forkhead box Q1; Sp1, specificity protein 1; SOX4, sex-determining region Y-box 4; TGFBR1, TGF-β receptor 1; CCNE2, cyclin E2; AKT, protein kinase B; CSCs, cancer stem cells; lncRNA, long non-coding RNA; LINP1, lncRNA in nonhomologous end joining (NHEJ) pathway 1; NF-κB, nuclear factor-kappa B; NKILA, NF-κB Interacting LncRNA.

## Abbreviations

ADAM12	a disintegrin and metalloprotease 12
AKT	protein kinase B
ATB	activated by TGF-β
CCNE2	cyclin E2
ceRNA	competing endogenous RNA
circRNA	circular RNA
Cox-2	cyclooxygenase-2
cPP1	catalytic subunit of protein phosphatase 1
CSC	cancer stem cell
CTC	circulating tumor cell
DAC	Decitabine
DAP12	DNAX activating protein 12 kDa
DDP	cisplatin
DOCK4	dedicator of cytokinesis 4
EC	endothelial cell
ECM	extracellular matrix
EED	embryonic ectoderm development
EMNs	exosome-mimetic nanosystems
EMT	epithelial-mesenchymal transition
ERK	extracellular signal-regulated kinase
EZH2	enhancer of zeste homologue 2
F-EMNs	Functionalization of EMNs with ITGα6β4
FOXM1	forkhead Box M1
FOXQ1	forkhead box Q1
HCP5	histocompatibility leukocyte antigen complex P5
HDAC4	histone deacetylase 4
HGAL	human germinal center-associated lymphoma
IL-10	interleukin-10
JAB1	c-Jun activation domain-binding protein 1
JAK	Janus-activated kinase
LATS2	large tumor suppressor homology 2
LEC	lymphatic endothelial cell
LINP1	lncRNA in nonhomologous end joining (NHEJ) pathway 1
LMO7	LIM-domain only protein 7
lncRNA	long non-coding RNA
MAGI2	membrane-associated guanylate kinase, WW and PDZ domain-containing protein 2
MALAT1	metastasis-associated lung adenocarcinoma transcript 1
MAPK	mitogen-activated protein kinase
MICA	major histocompatibility complex class I chain-related A
miRNA	microRNA
mRNA	messenger RNA
MMP	matrix metalloprotease
MRE	miRNA response element
MTDH	metadherin
MT1-MMP	membrane type 1 MMP
MYEOV	myeloma overexpressed gene
nc-RNA	non-coding RNA
NEDD4L	neural precursor cell expressed developmentally downregulated gene 4-like
NF-κB	nuclear factor-kappa B
NK	natural killer
NKILA	NF-κB Interacting LncRNA
NSCLC	non-small cell lung cancer
PAI-1	plasminogen activator inhibitor-1
PD-1	programmed cell death protein-1
PD-L1	programmed cell death ligand-1
pFAK	phosphorylated focal adhesion kinase
PGE2	prostaglandin E2
PI3K	phosphatidylinositol-3-kinase
PITX1	paired-like homeodomain 1
PRC2	polycomb repressive complex 2
PRKD3	protein kinase D 3
PTX	paclitaxel
qRT-PCR	quantitative real-time polymerase chain reaction
ROBO1	roundabout, axon guidance receptor, and homolog 1
SCLC	small cell lung cancer
SMURF	Smad ubiquitin regulatory factor
SOX4	sex-determining region Y-box 4
SOX7	SRY-Box 7
Sp1	specificity protein 1
STAT3	signal transducer and activator of transcription 3
SULF2	sulfatase 2
TBILA	TGF-β-induced lncRNA
TCF4	transcription factor 4
TGF-β	transforming growth factor-β
TGFBR	TGF-β receptor
TIF1γ	transcriptional intermediary factor 1 γ
Tregs	regulatory T cells
USP15	Ubiquitin specific protease 15
UTR	untranslated region
XIST	X inactivate-specific transcript
YAP	yes-associated protein
ZEB	Zinc finger E-box-binding homeobox
Zo-1	zonula occludens-1
